# Supplementary material for: Control of Brushless Direct-Current Motors Using Bioelectric EMG Signals
Source: Sensors (Basel). 2022 Sep 9;22(18):6829. doi: 10.3390/s22186829 (PMC9504870; doi:10.3390/s22186829)
Supplement: Supplementary file 1 [file sensors-22-06829-s001.zip › sensors-1880002-supplementary.pdf]

Supplementary S1. Diagram of data acquisition; communication handling circuit diagram; diagram of a symmetrical power supply circuit

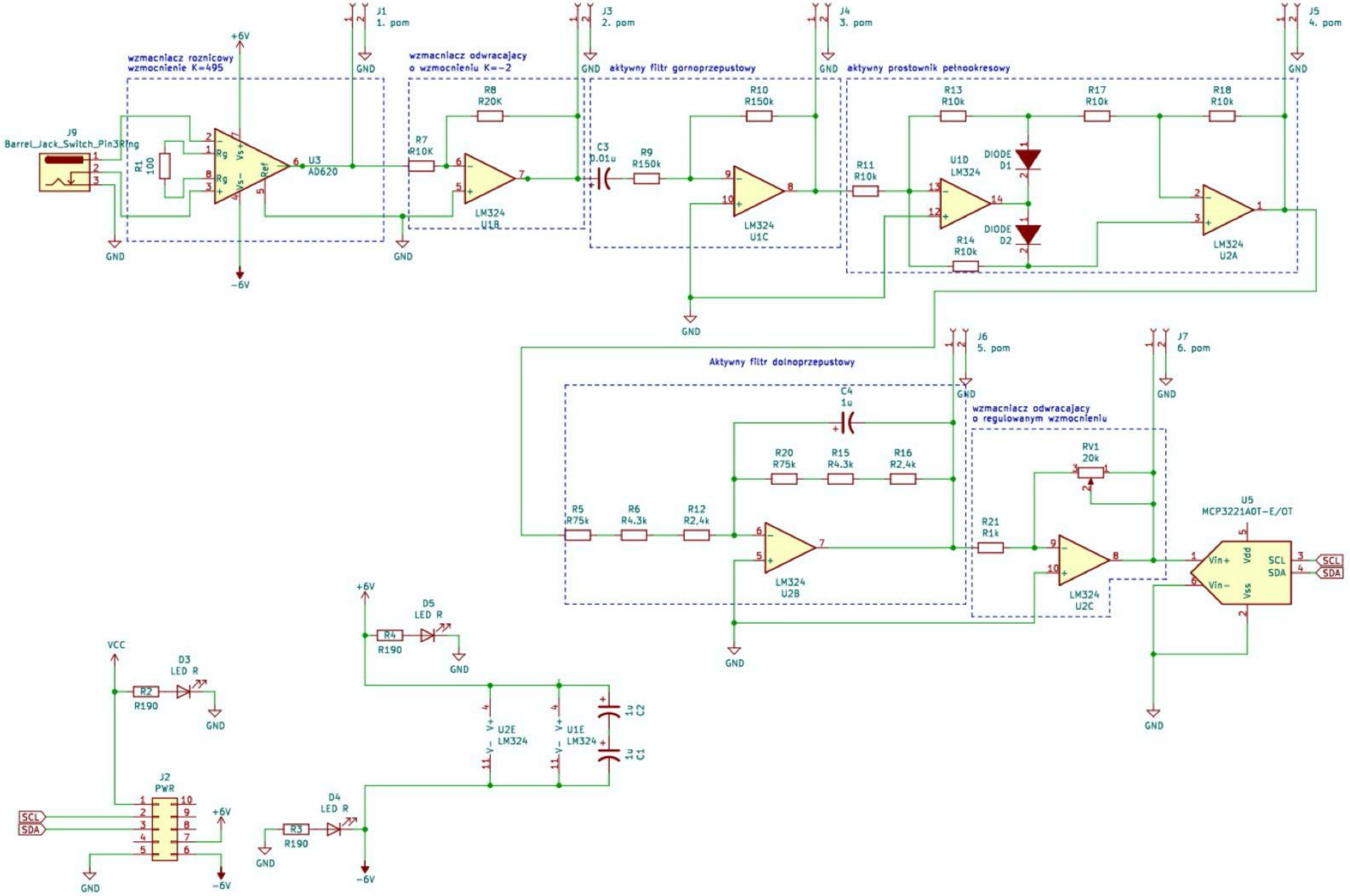

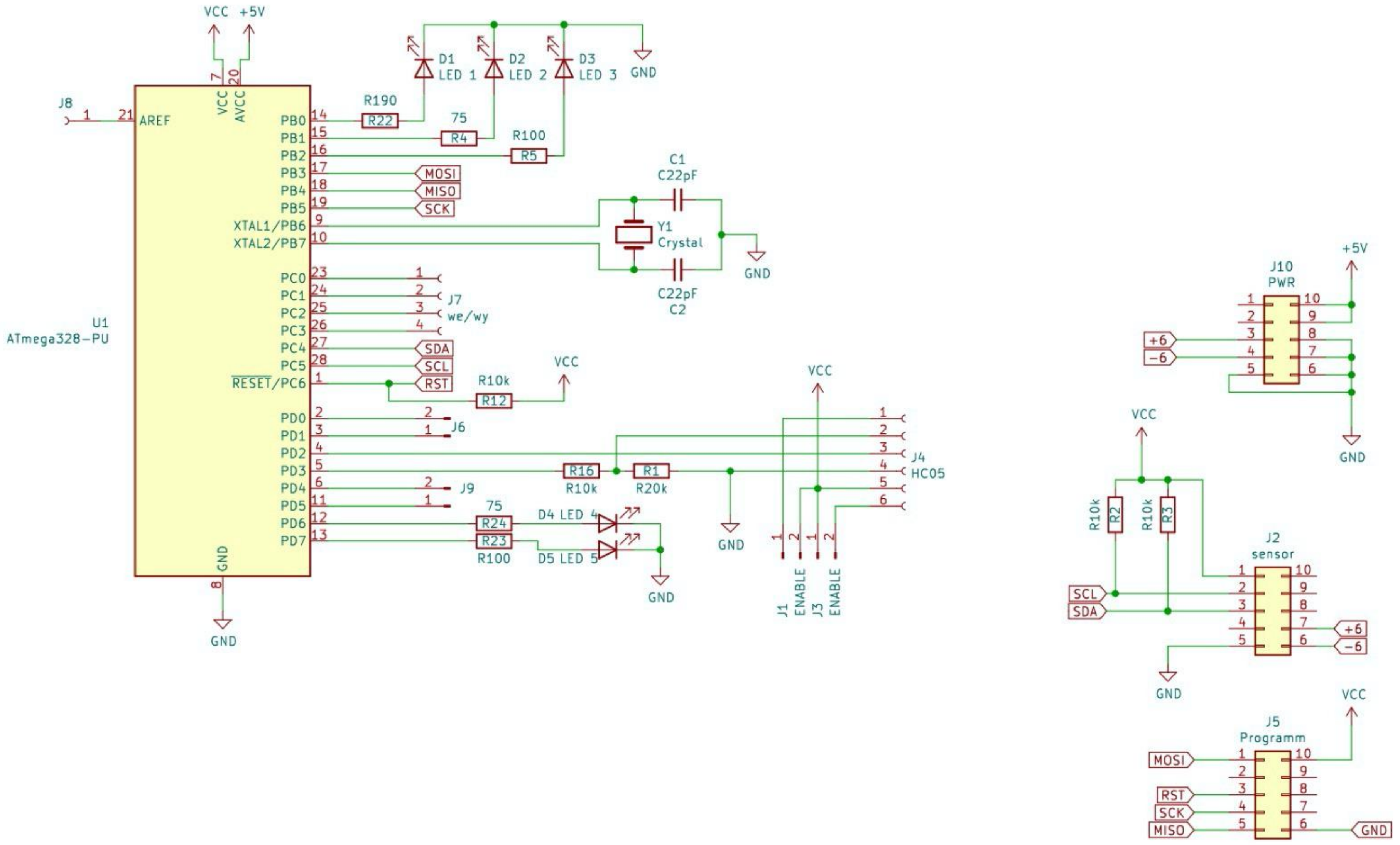
VCC
+5V
D1
LED 1
D2
LED 2
D3
LED 3
GND
J8
AREF
VCC
AVCC
R190
R22
75
R4
R100
R5
PB0
PB1
PB2
PB3
PB4
PB5
XTAL1/PB6
XTAL2/PB7
MOSI
MISO
SCK
C1
C22pF
Y1
Crystal
C22pF
C2
GND
PC0
PC1
PC2
PC3
PC4
PC5
RESET/PC6
J7
we/wy
SDA
SCL
RST
U1
ATmega328-PU
R10k
R12
VCC
PD0
PD1
PD2
PD3
PD4
PD5
PD6
PD7
J6
J9
VCC
J4
HC05
R16
R10k
R1
R20k
75
R24
R23
R100
D4 LED 4
D5 LED 5
GND
GND
J1
ENABLE
J3
ENABLE
GND
J10
PWR
+5V
+6
-6
GND
VCC
R10k
R2
R10k
R3
J2
sensor
SCL
SDA
+6
-6
GND
VCC
J5
Programm
MOSI
RST
SCK
MISO
GND

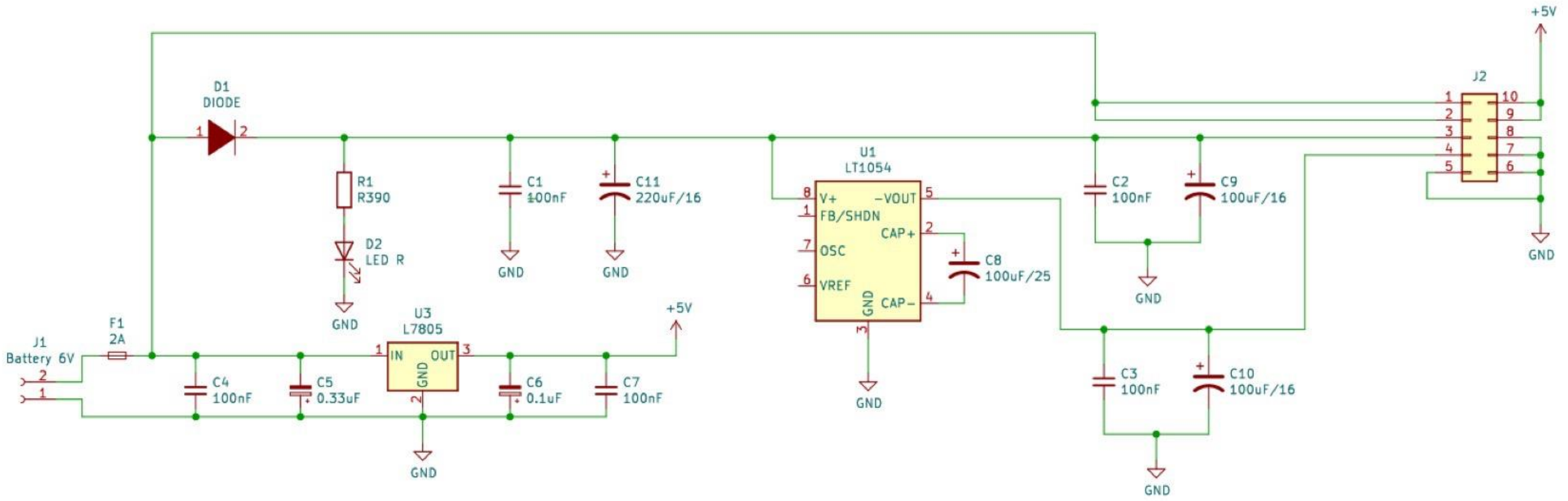
+5V
J2
D1
DIODE
U1
LT1054
R1
R390
C1
100nF
C11
220uF/16
V+
FB/SHDN
OSC
VREF
-VOUT
CAP+
CAP-
GND
C8
100uF/25
C2
100nF
C9
100uF/16
GND
D2
LED R
F1
2A
J1
Battery 6V
U3
L7805
IN
OUT
+5V
C4
100nF
C5
0.33uF
C6
0.1uF
C7
100nF
C3
100nF
C10
100uF/16

Supplementary S2. Diagrams of designed electric circuits of the BLDC motor control system made in the KiCad environment

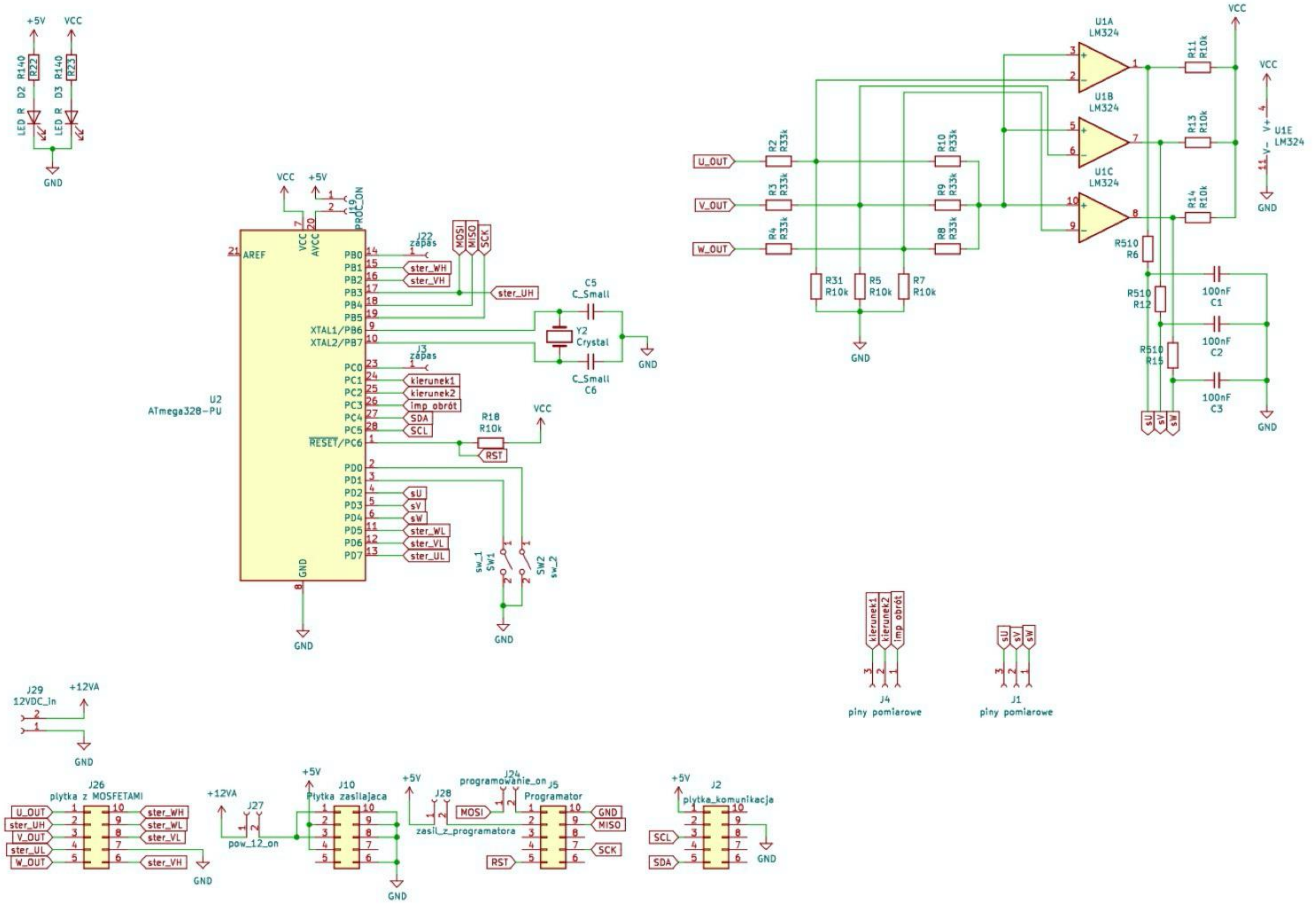

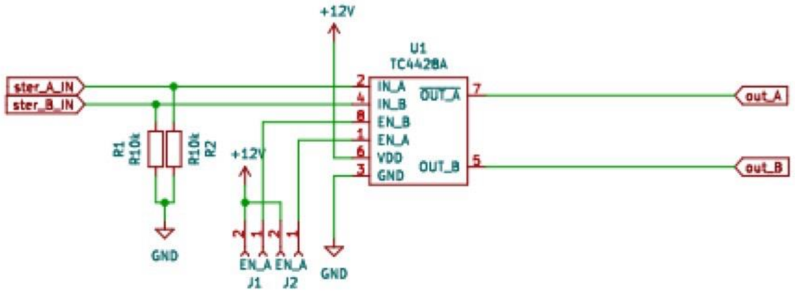
+12V
U1
TC4428A
ster_A_IN
ster_B_IN
IN_A
IN_B
EN_B
EN_A
VDD
GND
OUT_A
OUT_B
out_A
out_B
R1
R10k
R10k
R2
GND
+12V
EN_A
EN_A
J1
J2
GND

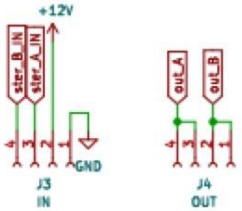
+12V
ster_B_IN
ster_A_IN
GND
J3
IN
out_A
out_B
J4
OUT

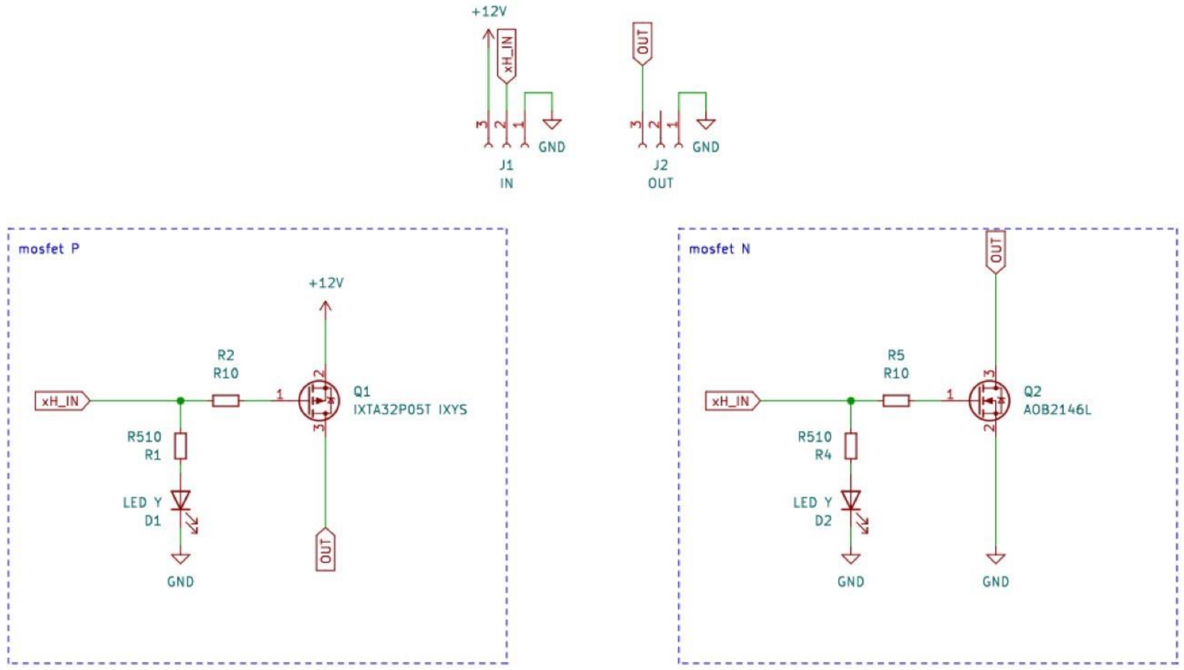
+12V
xH_IN
GND
J1
IN
OUT
GND
J2
OUT
mosfet P
+12V
R2
R10
xH_IN
Q1
IXTA32P05T IXYS
R510
R1
LED Y
D1
GND
OUT
mosfet N
OUT
R5
R10
xH_IN
Q2
AOB2146L
R510
R4
LED Y
D2
GND
GND

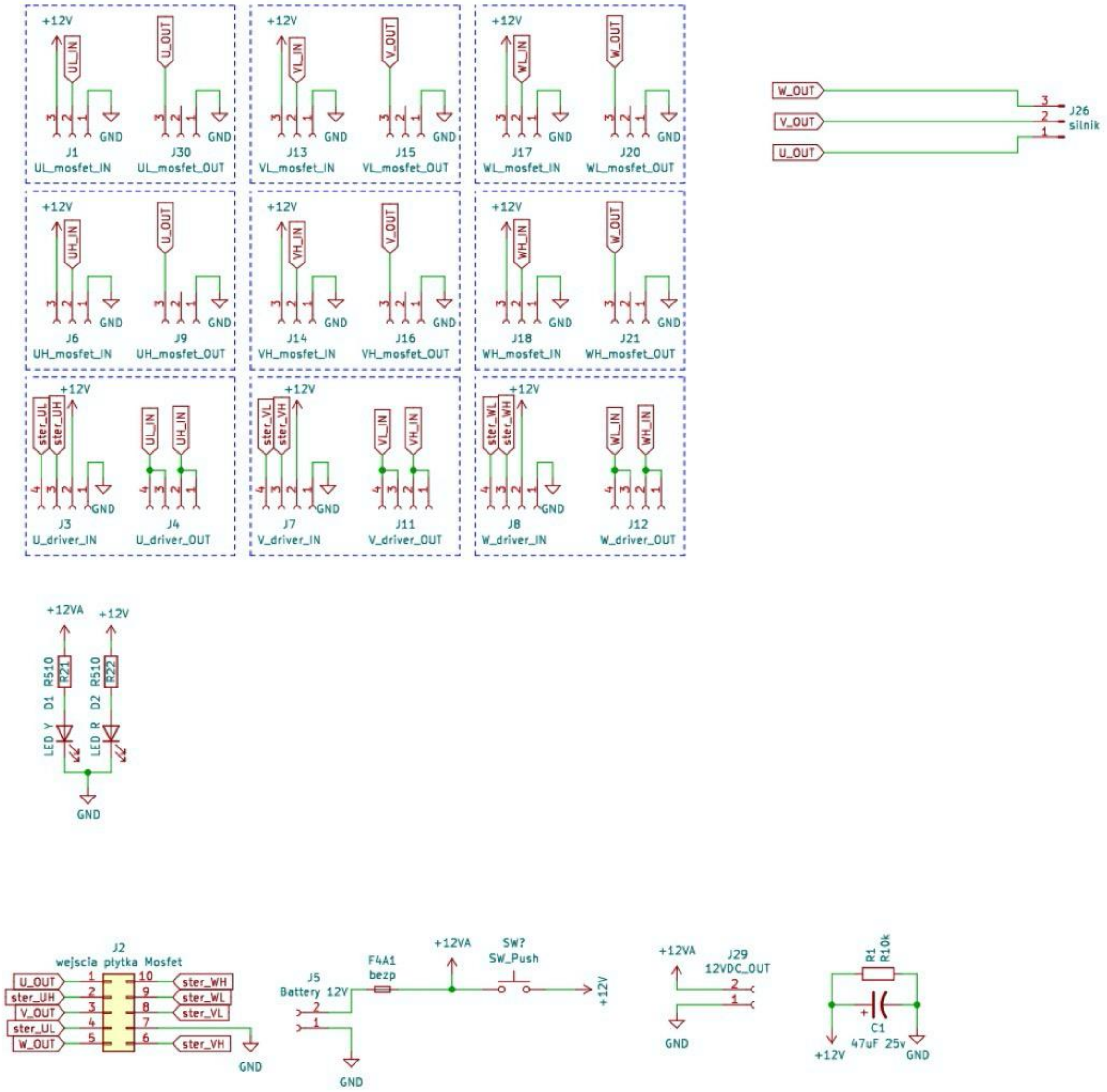
+12V
UL_IN
U_OUT
GND
J1
UL_mosfet_IN
J30
UL_mosfet_OUT
VL_IN
V_OUT
J13
VL_mosfet_IN
J15
VL_mosfet_OUT
WL_IN
W_OUT
J17
WL_mosfet_IN
J20
WL_mosfet_OUT
UH_IN
J6
UH_mosfet_IN
J9
UH_mosfet_OUT
VH_IN
J14
VH_mosfet_IN
J16
VH_mosfet_OUT
WH_IN
J18
WH_mosfet_IN
J21
WH_mosfet_OUT
ster_UL
ster_UH
J3
U_driver_IN
J4
U_driver_OUT
ster_VL
ster_VH
J7
V_driver_IN
J11
V_driver_OUT
ster_WL
ster_WH
J8
W_driver_IN
J12
W_driver_OUT
J26
silnik
+12VA
R510
R21
R22
D1
D2
LED Y
LED R
J2
wejscia płytka Mosfet
ster_UL
ster_VL
ster_WL
ster_WH
ster_VH
J5
Battery 12V
F4A1
bezp
SW?
SW_Push
J29
12VDC_OUT
R1
R10k
C1
47uF 25v
